# Spatial and temporal patterns of enzootic raccoon rabies adjusted for multiple covariates

**DOI:** 10.1186/1476-072X-6-14

**Published:** 2007-04-11

**Authors:** Sergio Recuenco, Millicent Eidson, Martin Kulldorff, Glen Johnson, Bryan Cherry

**Affiliations:** 1 School of Public Health, University at Albany-SUNY, One University Place, Rensselaer, New York, USA; 2 Zoonoses Program, Bureau of Communicable Disease Control, New York State Department of Health, 621 Corning Tower, Empire State Plaza, Albany, New York, USA; 3 Department of Ambulatory Care and Prevention, Harvard Medical School, 133 Brookline Ave, 6^th ^floor, Boston, Massachusetts, USA

## Abstract

**Background:**

With the objective of identifying spatial and temporal patterns of enzootic raccoon variant rabies, a spatial scan statistic was utilized to search for significant terrestrial rabies clusters by year in New York State in 1997–2003. Cluster analyses were unadjusted for other factors, adjusted for covariates, and adjusted for covariates and large scale geographic variation (LSGV). Adjustments were intended to identify the unusual aggregations of cases given the expected distribution based on the observed locations.

**Results:**

Statistically significant clusters were identified particularly in the Albany, Finger Lakes, and South Hudson areas. The clusters were generally persistent in the Albany area, but demonstrated cyclical changes in rabies activity every few years in the other areas. Cluster adjustments allowed the discussion of possible causes for the high risk raccoon rabies areas identified.

**Conclusion:**

This study analyzed raccoon variant rabies spatial and temporal patterns in New York that have not been previously described at a focal (census tract) level. Comparisons across the type of spatial analysis performed with various degrees of adjustment allow consideration of the potential influence of geographical factors for raccoon rabies and possible reasons for the highest risk areas (statistically significant clusters).

## Background

Raccoon rabies is a disease that is prevalent in the eastern United States with impacts on other wildlife and domestic species, and poses a threat to the human population. Raccoon rabies has been present in New York State (NYS) since 1990 [1]. Raccoon rabies entered NYS from the south and spread out northward and eastward, reaching the northern part of the state by 1998 [2]. Almost all of NYS is now a large enzootic area (with the exception of Long Island and the Adirondack Mountains). Efforts to contain the spread of the epizootic have been conducted since 1995 in the western, north and northeastern sections of the state by building immune barriers with oral rabies vaccine (ORV) targeting of raccoons [3,4]. Although ORV programs continue in 10 NYS counties neighboring Canada [5], most of NYS has not received an ORV intervention to deter the raccoon rabies enzootic which has now been active for at least a decade in most areas. In an enzootic area the lethal effect of rabies usually reduces the population of the reservoir species. Rabies activity increases when the area is repopulated by new generations of susceptible hosts, creating cycles with peaks every few years. These raccoon rabies cycles are reflected in the raccoon rabies incidence oscillations recorded by the NYS Department of Health (NYSDOH) rabies surveillance system at the town and county level [6,7].

The first ORV intervention in a NYS enzootic area was reported in small adjacent areas of Albany and Rensselaer counties from 1994–1997. This pilot study demonstrated rabies suppression by ORV, but the research study was discontinued after 1997[8]. Discussion of whether or not to intervene with ORV in enzootic areas has been ongoing [9,10], but currently ORV has been primarily utilized in epizootic areas with immune barriers to contain rabies spread or to progressively isolate circumscribed epizootic areas [5]. The high cost of ORV interventions, especially for large areas [11], is an obstacle to considering large-scale applications of ORV to control enzootic raccoon rabies.

To develop better control strategies using ORV or other interventions for raccoon rabies enzootic areas, it is necessary to examine the disease patterns in space and time, with the goal of understanding how such patterns might support the development of more efficient rabies control strategies [12,13].

The large NYS rabies enzootic area provides a unique opportunity to study raccoon rabies spatial patterns with respect to the natural and man-made environment in order to help explain raccoon rabies epidemiology in space and time. Raccoon rabies in NYS has been documented with a well-established surveillance system conducted by NYSDOH, local health departments (LHD), and other agency partners. Key features of this surveillance system include statutory reporting requirements, free laboratory testing of rabies-suspect animals, and partial reimbursement to local health departments for the cost of submitting animal specimens for testing. Available data include animal case reports, human exposure/incidents, human post-exposure treatments, cost of preventive activities, and laboratory test results. Rabies information from NYS has been utilized in national and regional rabies analyses, with data aggregated by town or county [14-16]. Recently, most of the terrestrial rabid animals reported to the NYSDOH have been geocoded to geographical coordinates, enabling the analysis of rabies patterns at a local level [17]. In this study, spatial and temporal patterns of the raccoon rabies epizootic in NYS are identified, and described with spatial cluster techniques, to assist in understanding the natural dynamics of raccoon rabies.

Factors associated with rabies geographical clustering may be identified by examining how clusters are modified after adjustment for geographic and human factors that may be associated with increased or decreased transmission. These may include land use type, land elevation, human population density, presence of major roads, presence of rivers/lakes, and protection from being adjacent to an ORV exposed area. Adjusting for those factors and for differences due to geographical location can assist us in identifying unusual groupings of raccoon rabies cases given the expected local distribution of observed raccoons.

## Results

Of the 4,671 terrestrial rabies cases included in the study, 2,974 (63.7%) were raccoons, 1,063 (22.8%) were skunks, and 634 (13.5%) were other animals including domestic and wildlife species. A review of the annual number of terrestrial animal tested for rabies from 1997 to 2003 in the 48 counties included in the study did not reveal systematic changes in surveillance efforts over time (Table 1).

**Table 1:** Annual number of terrestrial animals tested from 48 counties in the study area, New York, 1997–2003.

	**YEAR**						
	
**County**	**1997**	**1998**	**1999**	**2000**	**2001**	**2002**	**2003**
Albany	599	619	520	439	410	429	314
Allegany	53	35	32	47	49	35	40
Broome	107	115	73	120	104	99	80
Cattaraugus	76	62	55	59	67	59	45
Cayuga	271	184	173	160	130	121	83
Chemung	82	54	63	63	87	58	42
Chenango	53	55	39	27	33	24	37
Columbia	146	144	94	146	121	92	54
Cortland	202	139	110	111	80	71	55
Delaware	75	56	40	37	52	41	32
Dutchess	203	147	152	134	127	121	119
Erie	411	420	412	477	427	459	438
Fulton	19	14	15	11	14	16	18
Genesee	48	48	71	55	30	34	39
Greene	86	76	83	61	74	52	48
Hamilton	2	0	10	0	3	0	2
Herkimer	49	21	36	42	19	28	17
Lewis	107	55	36	45	50	41	41
Livingston	91	95	76	45	58	52	34
Madison	66	79	57	48	37	33	39
Monroe	92	122	132	100	99	104	89
Montgomery	47	31	26	30	25	21	18
Oneida	138	101	90	85	83	59	76
Onondaga	268	250	196	167	154	147	141
Ontario	140	91	78	74	80	50	65
Orange	157	157	152	144	124	125	145
Orleans	81	78	74	67	73	60	53
Oswego	118	139	101	93	86	93	56
Otsego	99	55	62	50	47	46	32
Putnam	43	45	50	34	48	44	45
Rensselaer	277	176	200	127	113	173	184
Rockland	168	129	122	122	111	110	87
Saratoga	241	129	137	84	98	85	84
Schenectady	134	106	103	102	97	78	73
Schoharie	56	39	35	37	46	38	49
Schuyler	34	23	30	22	23	22	32
Seneca	27	27	22	29	29	17	25
Steuben	106	103	103	74	92	65	80
Sullivan	73	78	62	40	27	34	37
Tioga	87	69	56	82	56	40	55
Tompkins	120	132	65	114	110	79	98
Ulster	161	191	188	117	115	136	122
Warren	42	44	44	47	33	26	34
Washington	107	102	86	64	54	75	59
Wayne	92	93	69	78	70	54	53
Westchester	465	412	282	385	390	312	267
Wyoming	31	43	40	39	45	39	32
Yates	54	51	44	47	41	35	26
Total	6,204	5,434	4,796	4,581	4,341	4,032	3,694

Source: Rabies Laboratories-Wadsworth Center, NYSDOH.

**Figure 1:**
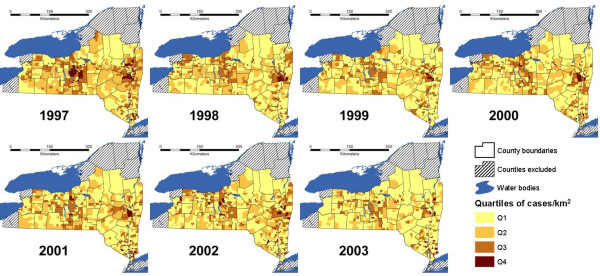
Terrestrial rabies cases per km^2 ^by year in quartile strata at census tract level, New York, 1997–2003.

The distribution of terrestrial rabid animals by year at the census tract level is presented in Figure 1. Grouping the census tracts in quartiles every year, the ones with the highest number of reported cases per km^2 ^were located mainly in the eastern edge (Hudson Valley), in the center (Finger Lakes region), and northwest of the study area.

**Figure 2:**
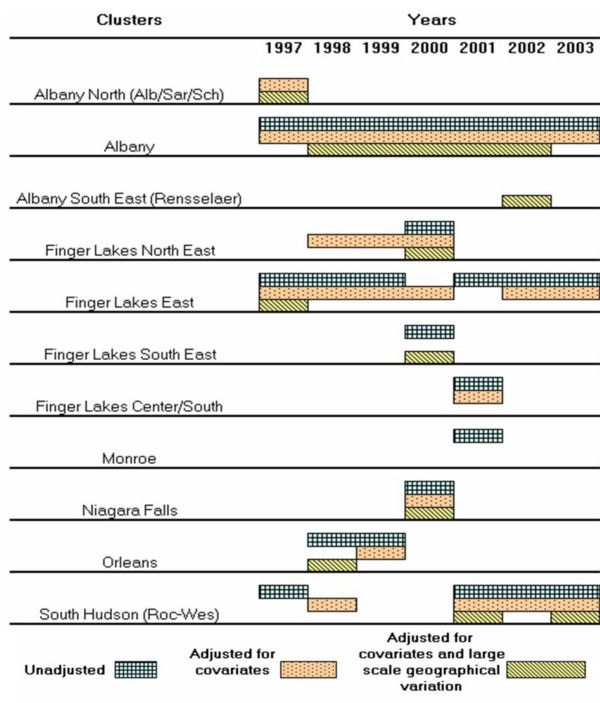
Statistically significant terrestrial rabies clusters, New York, 1997–2003, by type of purely spatial cluster analysis.

Table 2 summarizes the statistically significant (p ≤ 0.05) clusters by year and region for the three types of cluster analyses: unadjusted for covariates or large scale geographical variation (LSGV), adjusted for covariates but not for LSGV, and adjusted for covariates and LSGV. These clusters are summarized with a timeline in Figure 2.

**Table 2:** Location of statistically significant (p ≤ 0.05) terrestrial rabies clusters, New York, 1997–2003. Clusters are shown for models unadjusted for covariates or large scale geographical variation, adjusted for covariates, and adjusted for covariates and large scale geographical variation. Statistically nonsignificant clusters observed in the same location as significant clusters are also shown. (Dash indicates no cluster found).

		**Unadjusted**			**Adjusted for covariates**			**Adjusted for covariates and large scale geographical variation**		
				
**Year**	**Cluster location**	**Cases observed**	**RR**	***p-value***	**Cases observed**	**RR**	***p-value***	**Cases observed**	**RR**	***p-value***
1997										
	Albany	203	6.81	≤0.001	112	4.8	≤0.001	28	2.24	0.418
	Albany North (Saratoga)	-	-	-	51	5.54	≤0.001	51	4.72	≤0.001
	Finger Lakes East	181	3.88	≤0.001	172	4.23	≤0.001	146	1.8	≤0.001
	South Hudson (Roc-Wes)	60	3.22	≤0.001	19	2.31	0.973	4	9.08	0.942
1998										
	Albany	172	14.98	≤0.001	148	5.65	≤0.001	117	2.24	≤0.001
	Finger Lakes East	107	2.86	≤0.001	97	2.62	≤0.001	6	7.68	0.470
	Finger Lakes North East	-	-	-	42	2.24	0.022	32	2.14	0.334
	Orleans	44	2.85	≤0.001	3	15.4	0.936	20	3.49	0.015
	South Hudson (Roc-Wes)	-	-	-	12	5.86	0.020	4	26.05	0.083
1999										
	Albany	80	46.06	≤0.001	102	7.81	≤0.001	80	2.3	≤0.001
	Finger Lakes East	89	2.41	≤0.001	28	7.32	≤0.001	3	1.09	0.998
	Finger Lakes North East	3	49.99	0.101	33	2.86	0.004	3	20.36	0.731
	Orleans	15	7.96	≤0.001	15	5.09	0.006	30	2.18	0.359
2000										
	Albany	100	15.1	≤0.001	90	6.8	≤0.001	24	4.67	≤0.001
	Finger Lakes North East	73	2.71	≤0.001	44	2.65	≤0.001	21	3.6	0.009
	Finger Lakes East	5	11.86	0.260	52	2.7	≤0.001	-	-	-
	Finger Lakes South East	21	3.63	0.008	-	-	-	22	3.32	0.015
	Niagara Falls	8	33.26	≤0.001	8	18.2	≤0.001	8	42.1	≤0.001
2001										
	Albany	95	21.25	≤0.001	93	9.31	≤0.001	33	3.45	≤0.001
	Finger Lakes East	68	6.4	≤0.001	-	-	-	-	-	-
	Finger Lakes Center/South	53	2.14	0.003	140	1.74	≤0.001	79	1.64	0.106
	Monroe	7	10.16	0.047	-	-	-	7	50.2	0.869
	South Hudson (Roc-Wes)	39	3.12	≤0.001	35	2.92	0.002	32	2.53	0.028
2002										
	Albany	81	7.18	≤0.001	39	6.77	≤0.001	13	6.63	0.002
	Albany South East (Rensselaer)	-	-	-	-	-	-	16	4.69	0.007
	Finger Lakes East	77	2.17	≤0.001	35	3.93	≤0.001	5	6.34	0.962
	South Hudson (Roc-Wes)	80	1.69	0.025	6	14.2	0.028	6	9.79	0.181
2003										
	Albany	34	6.42	≤0.001	24	3.18	0.007	-	-	-
	Finger Lakes East	48	2.41	0.002	43	2.61	≤0.001	14	2.86	0.813
	South Hudson (Roc-Wes)	68	3.23	≤0.001	33	2.55	0.009	71	2.04	0.002

**Cluster locations**: Albany: Albany County; Albany North: parts of Albany, Saratoga, Schenectady counties; Albany South East: parts of Rensselaer, Columbia counties; Finger Lakes North East: parts of Cayuga, Oswego, Wayne counties; Finger Lakes East: parts of Cayuga, Oswego, Wayne Counties; Finger Lakes South East: parts of Broome, Chemung, Tioga, Tompkins counties; Finger Lakes Center/South: parts of Broome, Cayuga, Cortland, Onondaga, Ontario, Schuyler, Seneca, Steuben, Tioga, Tompkins, Yates counties; Monroe: Monroe County; Niagara Falls: Grand Island, northwest of Erie County; Orleans: Orleans County; South Hudson: parts of Dutchess, Orange, Putnam, Rockland, Sullivan, Ulster, Westchester counties.

### Cluster analysis unadjusted for covariates or LSGV

**Figure 3:**
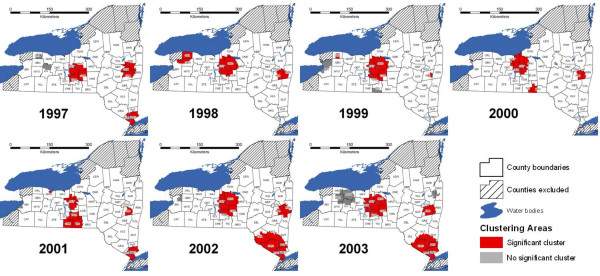
**Terrestrial rabies clusters, unadjusted for covariates or large scale geographical variation, New York, 1997–2003**. ***County abbreviations**: ALB: Albany; ALL: Allegany; BRO: Brooome; CAT: Cattaraugus; CAY: Cayuga; CHE: Chemung; CHN: Chenango; COL: Columbia; COR: Cortland; DEL: Delaware; DUT: Dutchess; ERI: Erie; FUL: Fulton; GEN: Genesee; GRE: Greene; HAM: Hamilton; HER: Herkimer; LEW: Lewis; LIV: Livingston; MAD: Madison; MON: Monroe; MNT: Montgomery; ONE: Oneida; ONO: Onondaga; ONT: Ontario; ORG: Orange; ORL: Orleans; OSG: Oswego; OTS: Otsego; PUT: Putnam; REN: Rensselaer; ROC: Rockland; SAR: Saratoga; SCH: Schenectady; SCR: Schoharie; SCY: Schuyler; SEN: Seneca; STE: Steuben; SUL: Sullivan; TIO: Tioga; TOM: Tompkins; ULS: Ulster; WAR: Warren; WAS: Washington; WAY: Wayne; WES: Westchester; WYO: Wyoming; YAT: Yates*.

In the cluster analysis unadjusted for covariates or LSGV, 3 to 5 statistically significant clusters were detected each year, for a total of 24 in the 7-year study period (Table 2). Albany County had statistically significant rabies clusters in all years, and consistently had the highest relative risk for a rabies cluster in most of the years. The persistence of the Albany County cluster can also be seen in the timeline summary of significant clusters (Figure 2). No other areas had persistent and significant clusters in the same location for all seven years of the study period (Table 2, Figures 2, 3). However, significant clustering was found in one or more locations of the Finger Lakes region (Broome, Cayuga, Cortland, Onondaga, Ontario, Oswego, Schuyler, Seneca, Steuben, Tioga, Tompkins, Yates, and Wayne counties) through the study period (Figure 3).

### Cluster analysis adjusted for covariates but not LSGV

Adjusting for covariates (land use type, land elevation, presence of major roads, presence of rivers/lakes, human population density, and protection from adjacent ORV exposed area), 24 significant clusters were detected in the 7-year study period, with 3 to 4 clusters observed each year (Table 2). Albany County or its area had the highest risk of rabies clusters in alternate years (1997, 1999, 2001, and 2003). The cluster in the Niagara Falls area in 2000 was the area of highest relative risk (RR: 18.16, p ≤ 0.001).

**Figure 4:**
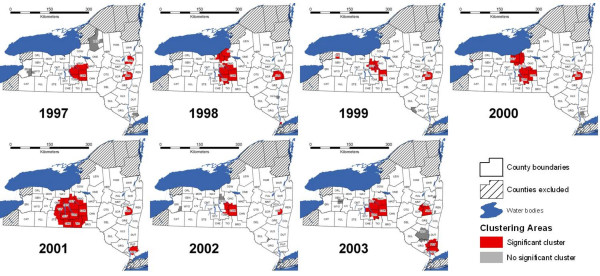
**Terrestrial rabies clusters adjusted for covariates, New York, 1997–2003**. *Covariates*: land use type, land elevation, presence of major roads, presence of rivers/lakes, human population density, and protection from adjacent ORV exposed area. ***County abbreviations**: ALB: Albany; ALL: Allegany; BRO: Brooome; CAT: Cattaraugus; CAY: Cayuga; CHE: Chemung; CHN: Chenango; COL: Columbia; COR: Cortland; DEL: Delaware; DUT: Dutchess; ERI: Erie; FUL: Fulton; GEN: Genesee; GRE: Greene; HAM: Hamilton; HER: Herkimer; LEW: Lewis; LIV: Livingston; MAD: Madison; MON: Monroe; MNT: Montgomery; ONE: Oneida; ONO: Onondaga; ONT: Ontario; ORG: Orange; ORL: Orleans; OSG: Oswego; OTS: Otsego; PUT: Putnam; REN: Rensselaer; ROC: Rockland; SAR: Saratoga; SCH: Schenectady; SCR: Schoharie; SCY: Schuyler; SEN: Seneca; STE: Steuben; SUL: Sullivan; TIO: Tioga; TOM: Tompkins; ULS: Ulster; WAR: Warren; WAS: Washington; WAY: Wayne; WES: Westchester; WYO: Wyoming; YAT: Yates*.

A statistically significant cluster of rabies in Albany County persisted with a similar size throughout most of the study period (Figures 2, 4). This cluster was smaller in 2002, but in 2003 was at its maximum size. The highest relative risk for this cluster occurred in 2002 (Table 2) when it was the smallest in size. Another cluster in the Albany region was observed in Saratoga County only in 1997.

Persistent statistically significant clusters adjusted for covariates occurred in the overall Finger Lakes region through the study period (Table 2, Figures 2, 4). A cluster in the Finger Lakes East area (in parts of Cayuga, Cortland, Onondaga, Seneca, Tompkins, and Wayne counties) in 1997 and 1998 was reduced in size by 3/4 in 1999. However, the cluster was back to its 1997–1998 size in 2000, and increased in size again to become a large rabies cluster covering most of the Finger Lakes region in 2001. This cluster was reduced in size again in the subsequent two years of the study period. Another significant cluster was observed in the Finger Lakes North East area (in parts of Cayuga and Wayne counties) in 1998 to 2000, and in 2002. This northern Finger Lakes cluster was located at the edge of Lake Ontario in 1998. By 1999, the cluster was located inland, but by 2000 it was located again at the edge of Lake Ontario. In 2002, this cluster reappeared inland in a smaller size than in previous years.

When adjusted for covariates, statistically significant rabies clusters were found in parts of the Southern region (Dutchess, Orange, Putnam, Rockland, Sullivan, Ulster, and Westchester counties) in four years of the study period (Table 2, Figures 2, 4). The significant cluster in the South Hudson area in 1998 appeared in a small portion of Westchester County in 1998, reappeared in a larger portion of the county in 2001, was reduced in size in 2002, and finally expanded in 2003 to its largest size of the study period, including Westchester and Putnam counties.

The Northwest region (Erie, Genesee, and Orleans counties) had two significant rabies clusters adjusted for covariates during the study period (Table 2, Figures 2, 4). In 1999 a small significant cluster was found in Orleans County, and in 2000 a small significant cluster was found in the Niagara Falls area of Erie County.

### Cluster analysis adjusted for covariates and LSGV

In the cluster analysis adjusted for covariates and LSGV, 14 significant clusters were detected in the 7-year study period, with 1 to 4 clusters observed each year (Table 2, Figure 2). The cluster with the highest relative risk was located in the Niagara Falls area of Erie County (RR: 42.1) in 2000.

**Figure 5:**
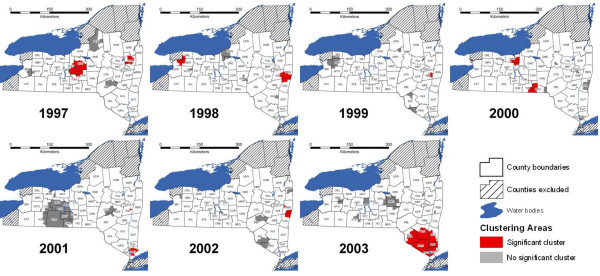
**Terrestrial rabies clusters adjusted for covariates and large scale geographical variation, New York, 1997–2003**. *Covariates*: land use type, land elevation, presence of major roads, presence of rivers/lakes, human population density, and protection from adjacent ORV exposed area. *Large scale geographical variation covariates*: county, ecoregion, and latitude. ***County abbreviations**: ALB: Albany; ALL: Allegany; BRO: Brooome; CAT: Cattaraugus; CAY: Cayuga; CHE: Chemung; CHN: Chenango; COL: Columbia; COR: Cortland; DEL: Delaware; DUT: Dutchess; ERI: Erie; FUL: Fulton; GEN: Genesee; GRE: Greene; HAM: Hamilton; HER: Herkimer; LEW: Lewis; LIV: Livingston; MAD: Madison; MON: Monroe; MNT: Montgomery; ONE: Oneida; ONO: Onondaga; ONT: Ontario; ORG: Orange; ORL: Orleans; OSG: Oswego; OTS: Otsego; PUT: Putnam; REN: Rensselaer; ROC: Rockland; SAR: Saratoga; SCH: Schenectady; SCR: Schoharie; SCY: Schuyler; SEN: Seneca; STE: Steuben; SUL: Sullivan; TIO: Tioga; TOM: Tompkins; ULS: Ulster; WAR: Warren; WAS: Washington; WAY: Wayne; WES: Westchester; WYO: Wyoming; YAT: Yates*.

The Albany region had three significant rabies clusters during the study period (Table 2, Figures 2, 5). A cluster in Saratoga County was found only in 1997. A cluster in Albany, Rensselaer, and Columbia counties that began in 1998 became smaller in 1999, and even smaller in 2000, continuing through 2002. In 2002, a significant cluster appeared at the eastern edge of the study area along the Rensselaer and Columbia county boundaries, where a part of the large Albany area significant cluster had been located in 1998.

The Finger Lakes region had statistically significant rabies clustering adjusted for covariates and large scale geographical variation in 1997 and 2000 (Table 2, Figures 2, 5). A cluster in the Finger Lakes East area was found in 1997. In 2000, two significant clusters were found, with one in Wayne County of the Finger Lakes North area, and another in Tioga and Broome counties of the Finger Lakes South East area.

The Southern region had a significant cluster of rabies in 2001, in Westchester County (Table 2, Figures 2, 5). A larger significant cluster reappeared in 2003, covering seven counties.

The Northwest region had a significant cluster in 1998 in Orleans, Genesee and Erie counties (Table 2, Figures 2, 5). Nonsignificant clustering was observed in that area in the subsequent year. In 2000 a small significant cluster appeared in Erie County of the Niagara Falls area.

### Comparison across types of cluster analyses

There are few differences in clustering in the Albany region depending on type of analysis, although persistent significant clustering is found for the unadjusted analyses and the analyses adjusted for covariates, whereas it does not occur at the beginning (1997) and end (2003) of the study period when adjusting for LSGV. This suggests that influence of the covariates and LSGV on the Albany region is small. In the Finger Lakes region the clustering is similar in the unadjusted analysis and the analysis adjusted for covariates. However, when adjusting for covariates and LSGV, the significant clustering disappeared in 1998, 1999, 2002 and 2003, suggesting that LSGV accounts for the clusters in those years. The Finger Lakes region has a unique land configuration in NYS, because the lakes divide the land into parallel valleys, acting as a natural barrier and keeping raccoon rabies movements within the valleys. Thus cases in this region appear less likely to build up to levels seen in other areas of the state, and unable to build up to larger significant enzootic outbreaks.

### Space-time permutation cluster analysis

**Figure 6:**
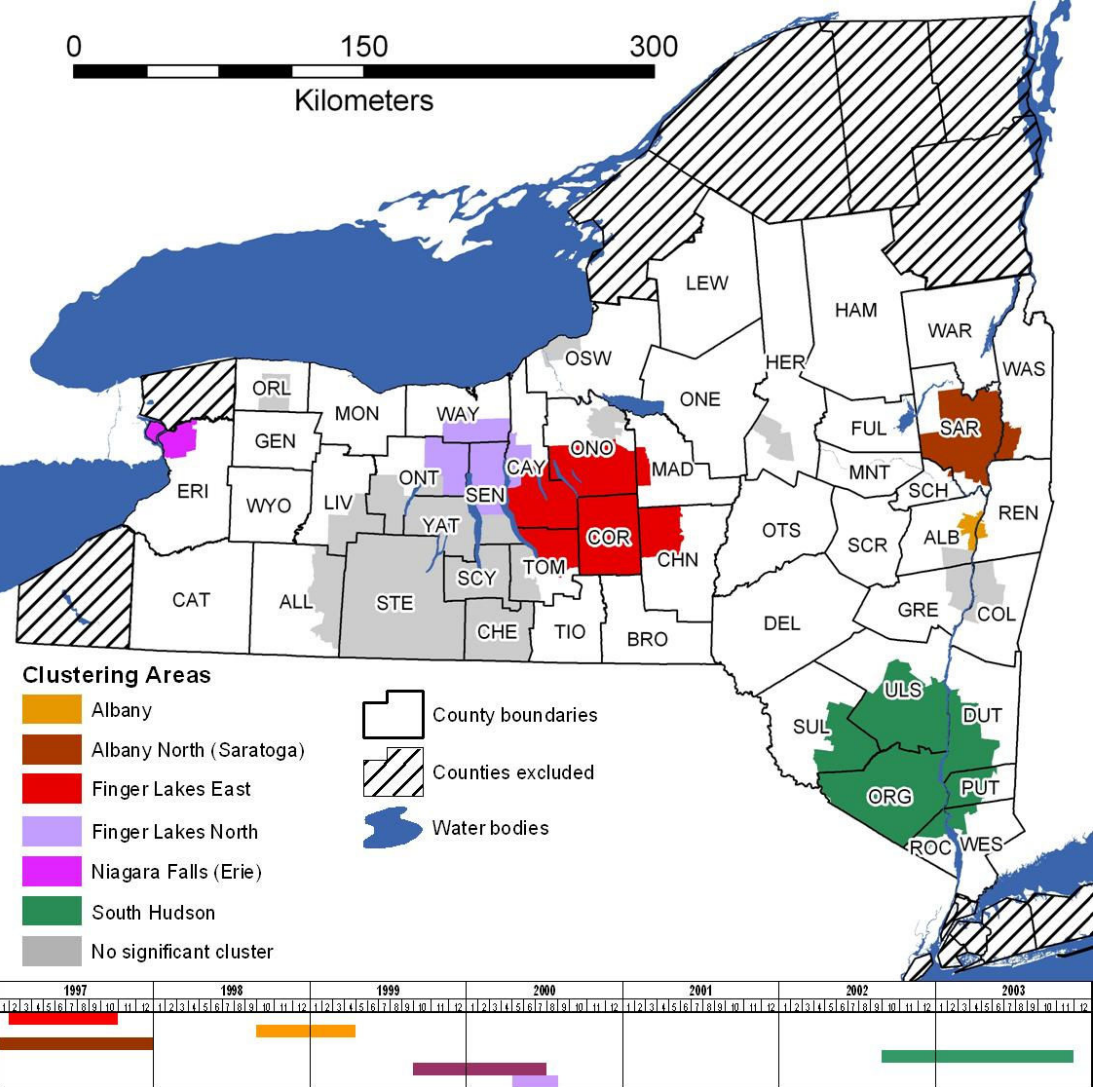
Location of statistically significant (p ≤ 0.05) terrestrial rabies clusters, New York, 1997–2003, for space-time permutation cluster search.

The cluster search with the space-time permutation approach detected six statistically significant clusters during the study period (Table 3). Most of the significant clusters occurred in the first half of the study period, indicating increased enzootic activity in Albany, Albany North (Saratoga County), Finger Lakes East, Finger Lakes North, and Niagara Falls areas (Figure 6). One cluster was identified in the South Hudson area at the end of the study period (2002–2003). This area was the only one with increased enzootic activity at the end of the study period. The average number of cases included in the clusters was 67.5 cases. The cluster including the largest number of rabies cases was located in the Finger Lakes East area in 1997, with 165 cases. The average duration of the identified clusters was 9.3 months. The cluster with the longest duration was located in the South Hudson area with 14 months (Table 3).

**Table 3:** Location of statistically significant (p ≤ 0.05) terrestrial rabies clusters, New York, 1997–2003, for space-time permutation cluster search.

**Cluster location**	**Time frame**	**Cases observed**	**RR**	***p-value***
Albany	10/1998 – 4/1999	48	3.09	≤0.001
Albany North (Saratoga)	1/1997 – 12/1997	81	2.13	≤0.001
Finger Lakes East	2/1997 – 10/1997	165	1.78	≤0.001
Finger Lakes North	5/2000 – 8/2000	25	3.76	0.014
Niagara Falls	10/1999 – 7/2000	16	6.04	0.008
South Hudson (Roc-W es)	10/2002 – 11/2003	70	2.13	0.006

## Discussion

Our analyses identified statistically significant clusters of raccoon rabies in specific areas of New York from 1997 to 2003. Those clusters were persistent in the Albany region for most of the study period in all three types of purely spatial analyses. Significant clustering was also found in one or more parts of the Finger Lakes region for most of the study period in all three types of analyses, although the location of the significant clustering varied more than in the Albany region. Clustering in the South Hudson region was present in 3–4 of the study years depending on type of analysis. Clustering in the northwest region of the state was more sporadic. The space-time cluster analysis demonstrated increased enzootic activity in the first half of the study period in northern and western areas of NYS, and at the end of the study the increased enzootic activity was concentrated in the South Hudson area.

This is the first study using the spatial scan statistic to identify terrestrial rabies clusters in an enzootic area at the census tract level. Spatial scan statistics investigate clustering above and beyond that anticipated by the adjustment factors [18-20]. Hence, without adjustment, we observe unusual aggregations of cases in different geographic areas. The adjustments for local land use, human population, etc, investigates how these unusual aggregations appear compared to factors related to raccoon habitat and the potential for human observation of sick raccoons. This allows us to identify the most unusual clusters of rabid animals given the expected local distribution of raccoons as observed by the surveillance system without any adjustments for covariates.

Although there are similarities in the size, distribution and location of some clusters in the unadjusted analyses with clusters in the adjusted analyses, the differences are worth noting. The unadjusted cluster analyses identify the areas of highest raccoon variant rabies reporting each year of the study period, and thus provide a valuable picture of the disease during 1997 to 2003. However, it is useful to determine whether significant geographical clustering of rabies cases occurs even after adjusting for geographic and human factors that may be associated with increased or decreased transmission and/or increased or decreased detection and reporting of a case, such as land use type, land elevation, human population density, presence of major roads, presence of rivers/lakes, and protection from being adjacent to an ORV exposed area. Using a Poisson regression model, a previous study in New York found that elevated numbers of raccoon-variant rabies cases in census tracts were associated with a higher proportion of low intensity residential areas (those with a lower concentration of housing units), lower land elevation, a lower proportion of wetlands, and a lack of rivers/lakes and major roads, after adjusting for LSGV [17]. Because raccoon rabies transmission occurs directly from animal to animal, terrestrial rabies cases are also by definition related spatially to one another, and thus have influence over the occurrence of rabies in the subsequent year. Thus, the use of expected values adjusted for LSGV (county, latitude, and ecoregion) is also important in identifying truly significant geographical clustering of rabies separate from this phenomenon.

The South Hudson area presented significant clusters in an apparent cycle in the unadjusted cluster analysis. A significant cluster in 1997 was followed by three years without clusters. Significant clustering reappeared in 2001 with increasing size in 2002 and 2003. A somewhat similar cycle was seen in the adjusted analyses, but the clusters were smaller in size and more often statistically nonsignificant. The presence of a large cluster in 2003 even when adjusting for covariates and LSGV indicates an increasing risk for raccoon rabies in the South Hudson area that may not be explained by those factors. The South Hudson region is highly populated by humans; however, population density was included as one of the covariates and thus cannot explain this large cluster. The South Hudson region borders the states of Connecticut to the east and New Jersey to the west, both states with current raccoon rabies enzootic activity. The rabies activity in the neighboring states may be influential in the South Hudson region cluster because that cluster area has a larger proportion of its boundary defined by other state edges than any other cluster area, and thus may be subject to an increased probability of influence from outside cases. However, such influence was not modeled in this study. It is also interesting to note that the South Hudson cluster preceded the first raccoon rabies epizootic observed in the neighboring region of Long Island in 2004.

The significant cluster found in 2001 with the unadjusted analyses in Rochester, Monroe County disappeared with the adjustment for covariates. With population density as one of the covariates, the cluster's disappearance may indicate that there was no significant clustering of cases beyond the association with people being available to report them. However, this phenomenon might be expected to impact reporting of clusters in other years, and none were reported.

Orleans County, another county in the northwestern region of the state, had clustering in 1997 that became statistically significant in 1998 and 1999 with the unadjusted analyses. There are some small differences in the appearance, size, and significance of clustering in the adjusted analyses, but no clear pattern. Orleans County borders an ORV exposed area in Niagara County and was chosen to be a control area (not exposed to ORV) for the ORV program for two years before the study period. There were no large changes in surveillance efforts for Orleans County during the study period. The cluster was located in the Iroquois National Wildlife Refuge, and this habitat may play a role in increasing raccoon rabies activity in the area. The cluster in the Niagara Falls area (Grand Island, Erie County), was very small and occurred only in 2000 in all three types of cluster analysis.

Interpretation of the nonsignificant clusters is difficult. Their presence in years before or after significant clusters was inconsistent. The presence of a nonsignificant cluster may indicate an increase in rabies activity that could be statistically significant the following year. However, the evidence found in this study is inconclusive regarding the utility of nonsignificant clusters as predictors of subsequent significant clusters.

The locations of the identified clusters using the space-time permutation approach were similar to the locations of the clusters identified using the Poisson model approach for the spatial scan statistic (purely spatial clusters). The space-time clusters demonstrated increased enzootic activity from 1997 to 2000, and identified the same foci of increased raccoon rabies activity at the end of the study period (South Hudson) as found by the purely spatial cluster analysis. Since these analyses automatically adjust for any purely spatial clusters, it should be noted that no area can have clusters during all years.

There are a number of additional factors that need to be considered in interpreting the results of these cluster analyses. The first factor is the potential influence of differential surveillance, which is always an issue when presenting unadjusted data. A strength of this approach is that the county component of the LSGV adjustment and the human population density component of the covariate adjustment help to specifically address potential differential surveillance. All states have rabies laboratories, and these adjustments are preferable to examining the unadjusted patterns of cases. After those adjustments, it is still possible for surveillance bias to occur due to differential surveillance in very small areas, for example, if a local outdoors club decides to collect animals for rabies testing in a focal area. We are not aware of any such focal surveillance efforts in the State. Although Albany County is the location for the state Rabies Laboratory and other federal agencies that participate in rabies surveillance and control, we have no evidence that this has led to increased surveillance in Albany or surrounding counties during this time period. There was increased surveillance by the wildlife laboratory for rabies in deer, but this effort was conducted statewide and in earlier time periods. The Finger Lakes area has the Cornell University Animal Health Diagnostic Center and College of Veterinary Medicine, although rabies testing is not available at those facilities. These agencies do not target surrounding towns and counties for increased rabies surveillance, but their location facilitates specimen transport and theoretically could increase public awareness. Areas with ORV that are targeted for increased surveillance were largely excluded from these cluster analyses, but Albany and Rensselaer counties were included even though small areas received ORV in early 1997 with increased active surveillance that year. It is possible that this could have influenced interest in specimen submissions in neighboring Saratoga County, which reported a significant rabies cluster only in 1997. Periodic significant clustering in the northwestern region of the State may have been influenced by an increased interest in surveillance due to the more active surveillance in neighboring ORV areas excluded from the study (the current ORV program in Niagara County began in 1997), although the variable appearance of these clusters by year is difficult to explain related to the consistent ORV work in that region. The authors participate in the NYS surveillance program, and are not aware of any specific changes in surveillance efforts in non-ORV areas through the study period. This conclusion was supported by an examination of the surveillance data by county and year (Table 1)

Surveillance may be influenced by other diseases in certain areas, such as distemper [21,22]. Distemper may result in an increase of dead animals, and the numbers of raccoons submitted for rabies testing. However, this increase in submissions due to dead animals with distemper would probably not increase the number of raccoons confirmed with rabies. Separate from any influence on surveillance, a distemper outbreak could decrease the chance of rabies transmission by decreasing the size of the raccoon population [22]. The presence and effect of other raccoon diseases could not be assessed in this study and may be a confounding factor to be considered in future research.

Because raccoon populations can be reduced by rabies with its ~100% case fatality rate, changes in the population are expected [21]. Once a rabies epizootic has occurred, raccoon and other impacted wildlife populations may need several years to rebound as the enzootic state is established, and will likely never reach the levels before rabies was introduced. Raccoon population changes can impact rabies cluster locations each year across the 7-year study period.

Misclassification of case location could have occurred, because addresses and geocoded coordinates can be subject to errors [23]. In addition, animals may move between the time of infection and the time of death, so the locations reported for the dead animals may not represent the locations of transmission [17]. However, we have no evidence that these potential location errors are systematic, and they should be minimized by the use of census tracts as the unit of analysis, as done in this study, rather than the specific geocoded address points.

Differences in human population density among census tracts influence sighting and reporting of dead animals or incidents where rabies may be suspected. Particularly in areas with very few people and high elevations, the lack of rabid animals could be due either to low raccoon densities at those elevations, or few people to see and report them. The inclusion of population density as a covariate helps to adjust for any differential reporting and minimize its influence in our results. However, these analyses will still primarily identify clusters of rabid animals that pose a risk to humans, which require control because of that risk.

## Conclusion

This study analyzed raccoon variant rabies spatial and temporal patterns in NY that have not been previously described at a focal (census tract) level. Comparisons across the type of spatial analysis performed (purely spatial cluster search unadjusted, adjusted for covariates, and adjusted for covariates and LSGV) allow consideration of the potential influence of geographical factors for raccoon rabies and possible reasons for the highest risk areas (statistically significant clusters). This approach is one of several to more fully understand areas of greatest risk for raccoon variant rabies, in order to better target potential ORV or other control programs [17]. Further research targeting these hotspots may help to refine the results and identify other factors that influence raccoon variant rabies in those areas.

Cluster areas identified with these types of analyses should be considered for raccoon rabies control interventions. Although rabies endemic cycles of approximately four years have been found in other analyses (7), in this study there is some overlap of clustering year-to-year, particularly in the Albany region. Prioritization for control based on clustering may be particularly valuable in areas such as the South Hudson region where the clustering is more compatible with an endemic cycle. This approach can even be done more frequently, e.g., in the spring and early summer of a year to prioritize areas for late summer and fall intervention. The areas of significant rabies clustering can be used as areas for piloting ORV programs for enzootic zones, especially when there are insufficient resources to develop an ORV program for an entire large enzootic region. Sections can be prioritized using the clustering areas as centers of each section. The areas for intervention can be prioritized for intervention considering size; number of cases observed in the cluster; recent clustering activity; and proximity to a current ORV program (to consider the area an extension of those ORV areas). The cluster areas could also be used in developing the borders for immune barriers to surround and progressively isolate the largest clustering areas. Other raccoon rabies prevention activities could also benefit from using the clustering areas identified. Public education on raccoon rabies exposures and the need for increasing pet vaccination activities may be prioritized in areas where clusters were identified.

It would be valuable to try this approach in areas that have received ORV. These areas would be very different from areas that have not experienced major vaccination campaigns, and so could not be included in the same study. However, this approach still may be useful at prioritizing areas within ORV zones, and should be examined in a future study.

When examining rabies patterns, adjustments are rarely made for differential surveillance or other factors that might influence the results. Thus, the approach of this study (adjusting for land use, elevation, human population, roads/rivers/lakes, and protection from being adjacent to an ORV area, and adjusting for large scale geographical variation by county, latitude, and ecoregion) is especially valuable in addressing any issues of differential surveillance by location or human factors.

## Methods

### Study area

The study area included the New York counties (excluding NYC and Long Island) that had not been exposed to ORV during the 1997–2003 study period. New York City does not participate in all aspects of the State's rabies surveillance program, and Long Island remained free of raccoon-variant rabies until 2004. The counties exposed to ORV programs during most of the study period were excluded, as indicated on the map in Figure 6. Those counties were Chautauqua, Clinton, Essex, Franklin, Jefferson, Niagara, and St. Lawrence. Counties exposed to ORV no more than once at the beginning (small parts of Albany and Rensselaer counties) or no more than twice at the end of the study period (Erie, Lewis, and Oswego counties) were not excluded. Because counties were included or excluded as a whole in the study, counties with small areas of ORV such as Oswego and Lewis were not excluded [24]. The selection criteria for the counties maximized the sample size for the raccoon rabies variant cases while keeping the study area comparable through the 7-year study period. Forty-eight counties were included in the study containing a total of 1,873 census tracts and 94,996.68 km^2 ^of land area.

### Data collection

The raccoon variant rabies cases were extracted from the geocoded rabies database of the Zoonoses Program, NYSDOH. This database was developed for a previous study, and included the geographical coordinates (latitude/longitude) of the addresses that were reported to the NYSDOH Wadsworth Center's Rabies Laboratory on its Rabies Specimen History form (DOH-487z) [17]. The forms are included with the rabies suspect samples submitted for testing. Data from 4,690 terrestrial animals confirmed with rabies from the study area during 1997 to 2003 were selected.

The cases selected for the study were assumed to be infected with raccoon rabies variant because ongoing variant testing by the Rabies Laboratory has confirmed raccoon variant in terrestrial animals during the study period (fox variant was reported in the early 1990's), and spillover from bats is very rare (14 cases in 20 years).^1 ^Terrestrial animals confirmed with bat rabies variants were excluded. To increase the number of cases in the study and maximize the statistical power of the study, the addresses of any terrestrial rabies cases that were not previously geocoded to a street level were processed to obtain geographical coordinates at a zipcode level or better with commercial software (MapMarker Plus 10.2™ by MapInfo Corporation). After geocoding, 4,671 cases were included in the study and 19 cases were excluded because the zipcode could not be determined. The cases were assigned to the corresponding census tract using a geographic information system (GIS) developed with ArcView 8.3™, and the analyses were performed at the census tract level of resolution. Census tracts are a universal unit of geography throughout the U.S., with covariate data available, to allow for generalizability of the approach to other regions.

### Cluster analysis

In this study a spatial scan statistic was utilized to detect statistically significant clusters of terrestrial rabies cases. This method has been previously utilized for research and surveillance of other zoonotic diseases [25-29]. The spatial scan statistic uses a circular moving window (purely spatial cluster search) or a cylinder window (space-time cluster search) that goes from one census tract centroid to another across the study area, increasing its size from zero to a maximum size specified by the user. The method finds the cluster that maximizes a likelihood function based on the Poisson distributions. Secondary clusters are also reported if they do not overlap with another reported cluster with higher likelihood. A p-value for each cluster is obtained using Monte Carlo hypothesis testing [30]. Calculations were done using the SaTScan™ v. 5.1.3 software [31].

Cluster analyses were conducted using census tracts as the unit of analysis. Purely spatial analysis was performed, scanning for clusters with high risks using the Poisson probability model [18], which requires cases and population counts within each potential cluster. Because raccoon and wildlife population counts or estimations are not available, the area of each census tract was used in lieu of population. Additionally, the number of rabies cases was adjusted for landscape covariates, which were used as a proxy for raccoon habitat and human-raccoon interactions. To apply the Poisson model we assumed under the null hypothesis that the number of raccoon rabies cases in a tract follows a Poisson distribution and the number of cases in a census tract is proportional to the census tract area. The size of the scanning window in the spatial scan statistic was allowed to increase until a maximum of 25% of the study area was reached. The statistical significance of the clusters was established using Monte Carlo hypothesis testing [30], by comparing the calculated likelihood ratio of each cluster to 999 Monte Carlo replications of the null distribution of the observed maximum likelihood ratio where cases are assumed to be randomly distributed across space. A cluster is considered statistically significant when its *p-value *was equal to or less than 0.05. Analyses were conducted separately for each year in the 7-year study period.

With the objective of observing the effect on rabies spatial clustering when some factors associated with raccoon variant rabies are controlled, we conducted cluster analyses adjusting for covariates. A previous study developed a Poisson regression model for factors associated with raccoon variant rabies in NYS [17]. In that model the dependent variable was the number of terrestrial rabies cases in a census tract and the independent variables were proportion of land use type (water, agricultural, high density residential, low density residential, commercial/industrial/transportation, barren, wetlands and forest) in a census tract, land elevation, human population density, presence of major roads in the census tract, presence of rivers/lakes in a census tract, and protection from being adjacent to an ORV exposed area. The model was also adjusted for county, latitude, and ecoregion to help adjust for possible unknown variables that co-vary spatially with the response across somewhat large geographic regions. Such variability is termed large scale geographical variation (LSGV) in our study – not to be confused with large "map scale".

The LSGV adjustment was also explicitly included to address possible influences in surveillance due to institutions such as the state Rabies Laboratory in Albany County and the veterinary college in the Finger Lakes region. There is no known reason that the veterinary college would have an influence on rabies surveillance, because it is not involved in specimen collection in any way. The Rabies Laboratory in Albany County could theoretically influence specimen collection in that specimens do not need to be shipped, but instead can be driven, from areas of Albany County and surrounding counties. There is no knowledge from the laboratory, state health department, or these counties that this has led them to increased surveillance. The state wildlife pathology laboratory is also located in Albany County. It is known that in previous years, special efforts were made for increased surveillance of rabid deer, for example, although these special studies did not occur during the study time period.

This Poisson regression model was utilized for our study area and the parameters obtained were used to calculate the expected number of terrestrial rabies cases in each census tract. The expected values were calculated for a model with the covariates only, and for a model adjusted for covariates and LSGV. The Poisson regression models were performed using SAS 9.1, with PROC GENMOD [32]. To obtain raccoon rabies clusters adjusted for covariates, the cluster analyses were repeated, replacing the census tract area values in the spatial scan statistic with the expected number of raccoon rabies cases obtained from the Poisson regression model [19]. Cluster searches were repeated utilizing the expected values adjusted for associated covariates alone and the expected values adjusted for covariates and LSGV.

An additional space-time cluster analysis was performed using a space-time permutation scan statistic [20,31]. This approach is a recent feature of SaTScan that requires only cases, allowing for cluster analysis in the absence of population data. The space-time permutation cluster analysis automatically adjusts for any purely spatial or purely temporal clusters, looking instead for clusters due to space-time interaction. It was used to search for increases in enzootic activity across the study area during the seven-year study period. This cluster search was retrospective, with the space unit represented by census tracts and the time unit represented by months. Because purely spatial clusters sustained in the same area over a number of consecutive years need not have the same radius whereas the cylinder-based space-time approach requires a constant radius, the two approaches might very well detect slightly different space-time clusters.

## Competing interests

The author(s) declare that they have no competing interests.

## Authors' contributions

SR and MK developed the spatial analyses. SR performed the analyses. All authors participated in the interpretation of the results and in the reviewing and approval of the final version of the article.
